# Optimising first- and second-line treatment strategies for untreated major depressive disorder — the SUN☺D study: a pragmatic, multi-centre, assessor-blinded randomised controlled trial

**DOI:** 10.1186/s12916-018-1096-5

**Published:** 2018-07-11

**Authors:** Tadashi Kato, Toshi A. Furukawa, Akio Mantani, Ken’ichi Kurata, Hajime Kubouchi, Susumu Hirota, Hirotoshi Sato, Kazuyuki Sugishita, Bun Chino, Kahori Itoh, Yoshio Ikeda, Yoshihiro Shinagawa, Masaki Kondo, Yasumasa Okamoto, Hirokazu Fujita, Motomu Suga, Shingo Yasumoto, Naohisa Tsujino, Takeshi Inoue, Noboru Fujise, Tatsuo Akechi, Mitsuhiko Yamada, Shinji Shimodera, Norio Watanabe, Masatoshi Inagaki, Kazuhira Miki, Yusuke Ogawa, Nozomi Takeshima, Yu Hayasaka, Aran Tajika, Kiyomi Shinohara, Naohiro Yonemoto, Shiro Tanaka, Qi Zhou, Gordon H. Guyatt, for the SUN☺D Investigators

**Affiliations:** 1 Aratama Kokorono Clinic, Nagoya, Japan; 2 Department of Health Promotion of Human Behavior, Kyoto University Graduate School of Medicine / School of Public Health, Yoshida Konoe-cho, Sakyo-ku, Kyoto, 606-8501 Japan; 3 Mantani Mental Clinic, Hiroshima, Japan; 4 Kabe Mental Health Clinic, Hiroshima, Japan; 5 Kokokara Clinic, Kochi, Japan; 6 Hirota Clinic, Kurume, Japan; 7 Harimayabashi Clinic, Kochi, Japan; 8 Oji Mental Clinic, Tokyo, Japan; 9 Ginza Taimei Clinic, Tokyo, Japan; 10 Sinsapporo Mental Clinic, Sapporo, Japan; 11 Narumi Himawari Clinic, Nagoya, Japan; 12 Shiki Clinic, Nagoya, Japan; 13 Department of Psychiatry and Cognitive-Behavioral Medicine, Nagoya City University Graduate School of Medical Sciences, Nagoya, Japan; 14 Department of Neuropsychiatry, Hiroshima University Graduate School of Biomedical & Health Sciences, Hiroshima, Japan; 15 Center to Promote Creativity in Medical Education, Kochi Medical School, Kochi University, Nankoku, Japan; 16 Department of Neuropsychiatry, University of Tokyo Hospital, Tokyo, Japan; 17 Department of Neuropsychiatry, Kurume University Medical School, Kurume, Japan; 18 Department of Psychiatry, Toho University School of Medicine, Tokyo, Japan; 19 Department of Neuropsychiatry, Tokyo Medical University, Tokyo, Japan; 20 Department of Neuropsychiatry, Kumamoto University Graduate School of Medicine, Kumamoto, Japan; 21 Department of Neuropsychopharmacology, National Center of Neurology and Psychiatry, Tokyo, Japan; 22 Department of Neuropsychiatry, Okayama University Hospital, Okayama, Japan; 23 Miki Mental Clinic, Yokohama, Japan; 24 Department of Biostatistics, Kyoto University Graduate School of Medicine / School of Public Health, Kyoto, Japan; 25 Department of Clinical Biostatistics, Kyoto University Graduate School of Medicine / School of Public Health, Kyoto, Japan; 26 Departments of Clinical Epidemiology and Biostatistics and of Medicine, McMaster University, Hamilton, Canada

**Keywords:** Major depressive disorder, Antidepressive agents: first-line treatment, Second-line treatment, Randomised controlled trial

## Abstract

**Background:**

For patients starting treatment for depression, current guidelines recommend titrating the antidepressant dosage to the maximum of the licenced range if tolerated. When patients do not achieve remission within several weeks, recommendations include adding or switching to another antidepressant. However, the relative merits of these guideline strategies remain unestablished.

**Methods:**

This multi-centre, open-label, assessor-blinded, pragmatic trial involved two steps. Step 1 used open-cluster randomisation, allocating clinics into those titrating sertraline up to 50 mg/day or 100 mg/day by week 3. Step 2 used central randomisation to allocate patients who did not remit after 3 weeks of treatment to continue sertraline, to add mirtazapine or to switch to mirtazapine. The primary outcome was depression severity measured with the Patient Health Questionnaire-9 (PHQ-9) (scores between 0 and 27; higher scores, greater depression) at week 9. We applied mixed-model repeated-measures analysis adjusted for key baseline covariates.

**Results:**

Between December 2010 and March 2015, we recruited 2011 participants with hitherto untreated major depression at 48 clinics in Japan. In step 1, 970 participants were allocated to the 50 mg/day and 1041 to the 100 mg/day arms; 1927 (95.8%) provided primary outcomes. There was no statistically significant difference in the adjusted PHQ-9 score at week 9 between the 50 mg/day arm and the 100 mg/day arm (0.25 point, 95% confidence interval (CI), − 0.58 to 1.07, *P* = 0.55). Other outcomes proved similar in the two groups.

In step 2, 1646 participants not remitted by week 3 were randomised to continue sertraline (*n* = 551), to add mirtazapine (*n* = 537) or to switch to mirtazapine (*n* = 558): 1613 (98.0%) provided primary outcomes. At week 9, adding mirtazapine achieved a reduction in PHQ-9 scores of 0.99 point (0.43 to 1.55, *P* = 0.0012); switching achieved a reduction of 1.01 points (0.46 to 1.56, *P* = 0.0012), both relative to continuing sertraline. Combination increased the percentage of remission by 12.4% (6.1 to 19.0%) and switching by 8.4% (2.5 to 14.8%). There were no differences in adverse effects.

**Conclusions:**

In patients with new onset depression, we found no advantage of titrating sertraline to 100 mg vs 50 mg. Patients unremitted by week 3 gained a small benefit in reduction of depressive symptoms at week 9 by switching sertraline to mirtazapine or by adding mirtazapine.

**Trial registration:**

ClinicalTrials.gov, NCT01109693. Registered on 23 April 2010.

**Electronic supplementary material:**

The online version of this article (10.1186/s12916-018-1096-5) contains supplementary material, which is available to authorized users.

## Background

Every year, an estimated five million people in high-income countries alone start new antidepressants to treat their depression [1–3]. In the USA, the annual prevalence of prescribed antidepressant use exceeds 10% of the population, almost double that of 10 years before [2]. Antidepressant use is similarly high and increasing in European countries, ranging between 4 and 9% [1], with a 1-year incidence of new antidepressant prescription of approximately 1% [3]. Clinicians need specific, detailed and appropriate guidelines to guide their antidepressant pharmacotherapy.

To initiate antidepressant treatment, modern guidelines recommend new generation antidepressants and in particular selective serotonin reuptake inhibitors (SSRIs) [4]. A network meta-analysis of 12 new generation antidepressants suggested that the SSRI sertraline, because of its favourable balance of benefits, acceptability and cost, may be the best choice when starting treatment for major depression [5]. Sertraline has been the most widely prescribed antidepressant in the USA [6] and elsewhere [7].

Once they choose a first-line antidepressant, practitioners must optimise its dosage, considering the wide approved dose range for most drugs. Many guidelines only list such ranges and do not specify where within this range the initial treatment should aim [4, 8, 9]. The American Psychiatric Association guideline is more specific and recommends that the initial doses be incrementally raised and maximised, side effects permitting ([10], p. 43). However, systematic reviews of randomised controlled trials (RCTs) have provided conflicting results regarding the benefits and harms of lower vs higher doses of various antidepressants within their therapeutic ranges. One review synthesised results from 33 RCTs comparing two or more doses of the same antidepressants and found that a dose level of 100–200 mg imipramine equivalents (or 20–40 mg fluoxetine equivalents) showed the highest response rate, while lower doses showed a reduction in efficacy and higher doses were not accompanied by increased efficacy. Adverse events increased monotonically with dose [11]. However, this study included both old and new generation antidepressants. The dose-response curves may be different for different classes of antidepressants. More recent reviews focussing on a single agent [12] or several agents [13] suggested that there was no dose-response relationship within the approved dose ranges for SSRIs.

Clinicians face another challenge because, after several weeks of the first-line treatment, only 50% of patients respond (i.e. achieve depression severity less than half that at pre-treatment), and only 30% remit (i.e. return to a euthymic state). Patients’ failure to respond or remit requires consideration of alternative treatments. Guideline recommendations for second-line treatment include dose escalation, switching to a different antidepressant or adding a different drug [4, 10]. The last strategy may be divided into ‘augmentation’ when a non-antidepressant drug is added to an antidepressant and ‘combination’ when two antidepressants are used together [4]. Systematic reviews of RCTs agree that dose escalation confers no benefit beyond continuing the initial drug dose [14–17]; consistent with these results, the German National Guideline clearly states that dose escalation for SSRI does not work [8]. Two previous systematic reviews have found some support for the switching strategy [18], especially to an antidepressant from a different class [19]. However, a recent more rigorous meta-analysis found no high-level evidence that switching the antidepressant is effective when compared to simply continuing the initial antidepressant [20]. There are also reviews that support various augmentation strategies [21] and combination strategies [22]. However, most of the RCTs addressing patients who fail to respond to initial pharmacotherapy for depression compared new strategies against continuing preceding treatment; far less evidence exists comparing alternative second-line strategies against one another. For example, only two reports have directly compared switching vs combination strategies among patients who failed first-line treatment: one study compared combination vs switching vs continuing the prior treatment [23], while another compared dose escalation vs combination vs continuing the prior treatment [24]. Unfortunately, neither study was sufficiently powered (only 32–38 patients in each arm in the first study and 98–99 patients in the second study) to reach meaningful conclusions. The STAR*D trial randomised 1439 patients with major depression who had not remitted on citalopram to various switching strategies or augmentation/combination strategies. However, they used an equipoise-stratified design which gave patients choices in their treatment regimen: only 105 patients consented to randomisation to any of the drug switching or augmentation strategies, and consequently the analyses had to be conducted separately among the switching strategies or among the augmentation/combination strategies [25, 26]. A recently published study compared switching to bupropion against combining with bupropion or augmenting with an antipsychotic aripiprazole among 1522 patients with antidepressant-refractory major depression and found that aripiprazole augmentation significantly increased response over bupropion combination or switching [27]. Most patients in this trial were, however, chronically depressed and highly refractory (the mean duration of index episode was more than 85 months and the mean number of previous medication courses was 2.4). The trial does not, therefore, address the initial treatment of a new depressive episode.

Even less is known about when to institute second-line treatment. The American Psychiatric Association practice guideline recommends 4–8 weeks [10], the American College of Physicians 6–8 weeks [28] and the National Institute for Clinical Excellence (NICE) guideline 3–4 weeks at one place but 6–8 weeks at another [4].

Thus, in trying to optimise antidepressant pharmacotherapy for patients with major depression, clinicians face conflicting guidelines informed by low quality evidence. We therefore conducted a pragmatic RCT — an RCT that mimics the practice environment and thus maximises applicability — to examine the following two questions concerning the first- and second-line treatments for a hitherto untreated episode of major depression:

1. What is the relative effectiveness and safety of initially titrating to the lowest vs the highest value of the therapeutic dose range of an antidepressant? On the basis of the results of prior evidence [5] and existing practices [6, 7], we chose sertraline as the drug to be tested. Since the available evidence suggests that there may be differences between different classes of antidepressants with respect to dose-response relationship [15], the first hypothesis pertains to the choice of the initial target dose of an SSRI, and of sertraline in particular, within its therapeutic range.
2. When patients do not remit on first-line treatment, what is the relative effectiveness and safety of continuing initial treatment, combining with mirtazapine or switching to mirtazapine? We set 3 weeks as this decision point as the earliest time point in guideline recommendations [4]. Our choice of mirtazapine is consistent with a systematic review reporting that combination of a reuptake inhibitor antidepressant such as an SSRI and a blocker of presynaptic alpha-2 autoreceptor (mirtazapine, mianserin, trazodone) was superior to other combinations [22]. In addition, mirtazapine poses a very low risk of drug interaction. Finally, in the recently updated network meta-analysis of 21 antidepressants, mirtazapine was the second most potent antidepressant after amitriptyline [29]; its less favourable acceptability profile renders it less suitable as first-line treatment, but when first-line treatment fails, mirtazapine represents a potentially appropriate second choice.

## Methods

### Study design and participants

The Strategic Use of New generation antidepressants for Depression (SUN☺D) is a multi-centre, open-label, assessor-blinded, pragmatic RCT that involved two randomisation steps to examine the first- and second-line treatment strategies for untreated unipolar major depressive episodes.

The study was conducted in the departments of psychiatry at 48 clinics and hospitals across Japan between December 2010 and September 2015. We recruited adult men and women between 25 and 75 years who had a primary diagnosis of a non-psychotic unipolar major depressive episode according to the Diagnostic and Statistical Manual of Mental Disorders, 4th Edition (DSM-IV) [30] within the past month as ascertained by the study psychiatrists administering the semi-structured interview using the major depression section of the Primary Care Evaluation of Mental Disorders (PRIME-MD) [31]. All recruited persons were also not allowed to be taking any antidepressant, antipsychotic or mood stabiliser. Other exclusion criteria included history of schizophrenia, schizoaffective disorder, bipolar disorder; current dementia, borderline personality disorder, eating disorder or substance dependence; and imminent high risk of suicide, as judged by the study psychiatrist. No severity threshold was required so long as the participant satisfied the diagnostic criteria for major depressive disorder in the past month. The protocol provides additional details of the eligibility criteria [32]. When the study psychiatrist decided that sertraline was indicated, the patient began taking 25 mg/day of sertraline; only those who tolerated sertraline for 3–16 days were eligible.

An institutional review board at each participating site approved the study. An independent data monitoring committee oversaw the trial. All participants provided written informed consent. The study protocol, its amendment and the statistical analysis plan have been published elsewhere [32, 33].

### Randomisation and masking

**Fig. 1:**
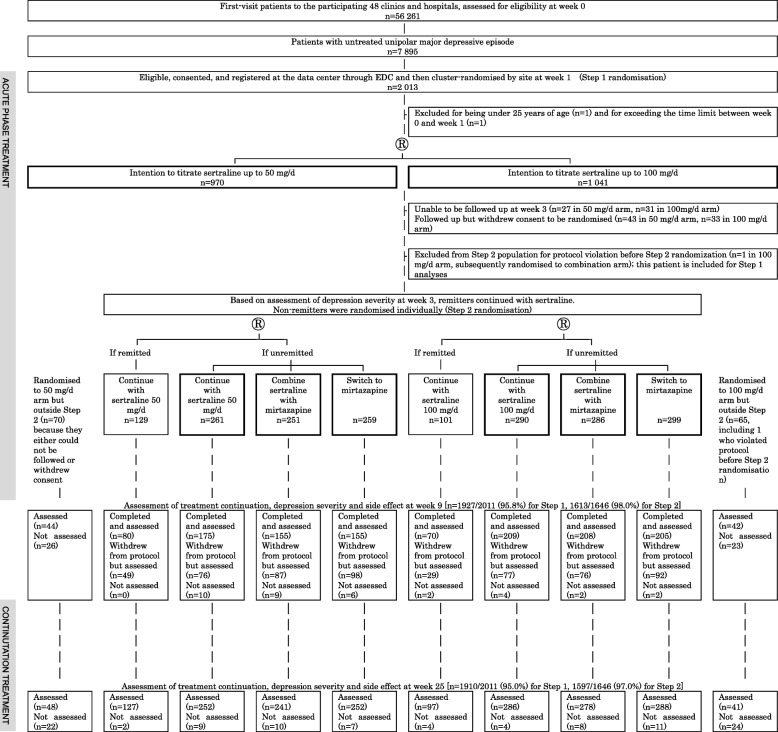
Trial profile. *®* randomised, *EDC* electronic data capture system

Figure 1 presents the study flow. In step 1, using the minimisation method adjusting for expected recruitment, study sites were randomised to titrate sertraline up to the minimum or the maximum of the licenced dosage in Japan (50 mg/day or 100 mg/day) by week 3. The unit of randomisation was therefore by site. The cluster randomisation design was chosen to avoid any confusion and possible protocol violation that might have arisen had we asked study physicians, who often had their own preference for either the minimum or the maximum target doses, to use different titrating strategies for different patients. Further, we were concerned that requiring patients to undergo two individual randomisations within 3 weeks might decrease the feasibility of this large pragmatic trial.

In step 2, participants who had not reached remission, defined as scoring 4 or less on the Patient Health Questionnaire (PHQ-9) [34] at week 3, were randomised 1:1:1 through the web-based central computer system to continue with sertraline, to combine sertraline with mirtazapine or to switch to mirtazapine. The step 2 randomisation used the minimisation method adjusting for site; whether 50% or greater reduction on PHQ-9 had been achieved; and whether patients reported moderate or greater impairment due to side effects.

Physicians and patients were aware of their treatments. Outcome assessors were blinded throughout. The statisticians conducting the analyses and the writing committee were masked to treatment allocation until they signed the agreed-upon interpretations of the results.

### Procedures

In step 1, participants in the minimum dosage arm received 50 mg/day of sertraline at week 1 to be continued through week 3, and those in the maximum dosage arm received 50–75 mg/day at week 1 and 100 mg/day at week 2 to be continued through week 3. When these target dosages were not tolerated, lower dosages were accepted.

In step 2, in the continuing sertraline arm, sertraline was administered as 50 or 100 mg/day according to the initial randomisation. In the combination arm, sertraline was continued and mirtazapine was added between 7.5 and 45 mg/day at the discretion of the study psychiatrist. In the mirtazapine switch arm, mirtazapine between 7.5 mg and 45 mg/day was administered; sertraline was tapered and discontinued by week 7.

Co-administration of benzodiazepines, but not of another antidepressant, antipsychotic or mood stabiliser was allowed up to week 9. Neither was any depression-specific psychotherapy such as cognitive behavioural therapy (CBT) or interpersonal psychotherapy (IPT) allowed. Those who withdrew consent to the study treatments by week 9 but still participated in follow-up evaluations received treatment as negotiated with their physician. Between weeks 9 and 25, there were no restrictions on treatments, and the continuation therapy was at the physician’s discretion.

### Outcomes

For both the step 1 and step 2 randomisations, the primary outcome was depression severity as measured by masked assessors conducting semi-structured interviews using the PHQ-9 at week 9. The PHQ-9 consists of the nine diagnostic criteria items of major depression from the DSM-IV [35]. Each item is rated between 0 = ’Not at all’ through 3 = ’Nearly every day’, making the total score range 0–27, with higher ratings indicating increased depression severity. The instrument has demonstrated excellent reliability, validity and responsiveness [34].

Secondary outcomes included the Frequency, Intensity and Burden of Side Effects Rating (FIBSER) and the Beck Depression Inventory 2^nd^ edition (BDI-II). The FIBSER collects information regarding the frequency, intensity and burden of side effects, each on a 7-point scale with higher ratings indicating greater severity, and provides a total score between 3 and 21 [25]. The BDI-II is a self-report measure of depression severity addressing 21 symptoms of depression, each on a scale between 0 and 3, with the total score ranging between 0 and 63; higher scores indicate more severe depression [36]. In addition, the study psychiatrist recorded any incident of suicidality according to the Columbia Classification Algorithm of Suicide Assessment (C-CASA) [37] or of manic/hypomanic/mixed episodes at weeks 9 and 25, and reported any serious adverse events throughout the study. Response was defined as 50% or greater reduction from week 1 on PHQ-9, and remission as scoring 4 or less on PHQ-9 [34].

The PHQ-9 and the FIBSER were administered by telephone assessors [38], trained at and operating from the central office and blind to the treatment assignment and the timing of the assessment, at weeks 1, 3, 9 and 25. We had previously established the high inter-rater reliability of our telephone assessors [39]. The BDI-II was filled in by the participants at weeks 0, 1 and then every 2 weeks up to week 9, and every 4 weeks up to week 25.

### Sample size

For step 1, we calculated that we would need 66 participants at each of 30 sites in order to ensure adequate power (beta level of 0.20) at a two-sided alpha level of 0.05 to detect a mean difference of 1 point on PHQ-9 with an estimated standard deviation (SD) of 5 or a standardised mean difference of 0.20 between the two arms, for the null hypothesis of no difference and an intracluster correlation coefficient of 0.05 [40]. We reasoned that an effect size of 0.20, corresponding with a small effect according to Cohen [41], would represent an important difference between active treatments. The required total sample size for step 1 was 1980.

For step 2, we calculated that 522 participants per group would ensure adequate power (beta level of 0.20) at a two-sided alpha level of 0.05 to detect a standardised mean difference of 0.20 among any two of the three treatment groups. Assuming a dropout rate of 10% and a remission rate of 10% at week 3, we needed 1934 participants entering step 1.

### Statistical analysis

In order to examine the optimum initial target dose of sertraline, we analysed results from all those who were cluster-randomised at week 1 to titrate sertraline up to 50 mg/day or 100 mg/day and, had they followed the protocol, would have stayed on that sertraline dose up to week 9 — that is, those who had remitted and continued on sertraline; those who had not remitted and were randomised at week 3 to continue sertraline; and those who declined the second randomisation. We used a weighted mixed-model repeated-measures analysis to compare model-adjusted least squares means: the primary outcome was the PHQ-9 at week 9 as pre-specified in the study protocol. The model included random effects for subjects and sites and fixed effects of treatment, visit (as categorical) and treatment-by-visit interaction, adjusted for age, sex, education, marital status, number of previous depressive episodes and baseline scores at weeks 0 and 1. At the first blinded interpretation committee meeting, the statistician reported an imbalance in the baseline demographic and clinical variables. The writing committee therefore agreed, at that meeting, without knowledge of the identity of the arms and based on their prior knowledge regarding variables that may be associated with depression severity, on the following variables to be entered in the primary analysis as possible confounders: age, sex, education, marital status, number of previous depressive episodes and baseline PHQ-9 scores at weeks 0 and 1. Each observation was weighted by inverse probability of censoring (IPCW) to account for missing outcomes due to being allocated to the combination or switching arms at step 2 randomisation. The weight for IPCW was calculated by a logistic regression that included age, sex, education, marriage, number of episodes, PHQ-9 scores at weeks 0 and 1 and whether the participant was allocated to continue sertraline or otherwise at week 3 as predictors of missingness. We applied the same modelling approach to the continuous secondary outcomes. We used weighted generalised linear mixed models with the logit link and binomial distributions to account for clustering effects for the dichotomous secondary outcomes. For the time to discontinuation of the allocated or any treatment, we applied Cox regression with the same covariates and calculated the hazard ratio (HR) and its 95% confidence interval (CI). For step 1 analyses, we examined two subgroup hypotheses, pre-specified in the statistical analysis plan [33]: (1) whether the PHQ-9 score at week 1 was less than or greater than 15 and (2) whether the patient had shown improvement from week 0 to week 1 at or above the median of the sample. We also conducted four sensitivity analyses employing mixed models with different assumptions, described in the statistical analysis plan [33].

To compare the second-line strategies, we used the mixed-model repeated-measures analysis comparing model-adjusted least squares means of the PHQ-9 at week 9 among the three treatment arms. This model included the following: fixed effects of PHQ-9 at week 3, treatment, visit (as categorical), treatment-by-visit interaction, minimisation variables for step 2 randomisation. The model included individuals and sites as random effects. We applied the same modelling approach to the continuous secondary outcomes and the logistic regression model for the dichotomous secondary outcomes. In order to facilitate the clinical interpretation of the obtained odds ratio (OR), we calculated the risk difference (RD) and its CI by applying the model-adjusted OR and its CI to the event rate in the sertraline continuation arm. For the time to discontinuation, we applied Cox regression with the same covariates. We applied the Hochberg procedure for adjusting the multiple comparisons involved with three treatment arms in the primary comparison and reported adjusted *P* values. For step 2 analyses, we examined three pre-specified hypotheses regarding possible effect modifiers [33]: (1) whether patients had achieved a 50% or greater reduction on PHQ-9 from week 1 to week 3, (2) whether FIBSER results indicated ’moderate’ or greater impairment due to side effects or (iii) whether step 1 was high or low dose.

All efficacy and safety analyses followed the intention-to-treat principle, and all patents were analysed in the groups to which they were randomised.

### Blinded interpretation of the results

The writing committee reviewed a statistical report in which the treatments were masked, and developed interpretation of the results and associated conclusions under alternative scenarios for all possible permutations of treatments [42]. The treatments were revealed only after the writing committee signed off on the agreed-upon interpretations (See Additional file 1).

### Role of the funding source

The funders of the study had no role in study design, data collection, data analysis, data interpretation or in writing of the report. The corresponding author had full access to all the data in the study and had final responsibility for the decision to submit for publication.

### Changes from the original protocol

No major change was made since the original protocol and statistical analysis plan [32, 33]. Our published protocol [32] lists the several minor changes in the details since the original protocol.

## Results

Figure 1 shows the screening, randomisation and follow-up of the study participants. Between December 2010 and March 2015, 56,261 first-visit patients to the participating 48 clinics and hospitals in Japan underwent eligibility assessment, of whom 7895 suffered from untreated unipolar major depressive episodes. Of these, 2011 patients satisfied eligibility criteria and were cluster randomised to titrate sertraline up to the minimum or maximum of the licenced dosage within 3 weeks: 970 in 22 clinics and hospitals to the 50 mg/day arm and 1041 in 26 clinics and hospitals to the 100 mg/day arm. The participants’ characteristics differed between the two arms, especially with regard to sex and PHQ-9 scores at weeks 0 and 1 (Table 1). Of those randomised at week 1, 1927 (95.8%) and 1910 (95.0%) were successfully followed up at week 9 and week 25, respectively.

**Table 1:** Baseline characteristics of the participants at step 1 randomisation

	Titrate sertraline up to 50 mg/day by week 3 (*n* = 970)	Titrate sertraline up to 100 mg/day by week 3 (*n* = 1041)
Demographic characteristics		
Age, year mean (SD)	43.3 (12.2)	41.8 (12.3)
Female sex, *n* (%)	572 (59.0)	506 (48.6)
Education year, mean (SD)	13.8 (2.2)	14.1 (2.5)
Job status, *n* (%)		
Employed full-time	398 (41.1)	374 (36.0)
Employed part-time	103 (10.6)	76 (7.3)
On medical leave	206 (21.3)	328 (31.6)
Housewife	114 (11.8)	116 (11.9)
Student	5 (0.5)	14 (1.4)
Retired	18 (1.9)	5 (0.5)
Not employed	124 (12.8)	125 (12.0)
Missing	2	3
Marital status, *n* (%)		
Single, never married	262 (27.1)	355 (34.1)
Single, divorced or separated	148 (15.3)	124 (11.9)
Single, widowed	29 (3.0)	25 (2.4)
Married	528 (54.6)	537 (51.6)
Missing	3	0
Clinical characteristics		
Age of onset at first episode, years, mean (SD)	38.6 (13.3)	37.1 (13.5)
Number of previous depressive episodes, mean (SD)	2.3 (4.2)	2.2 (3.1)
Length of current episode, months, mean (SD)	6.6 (17.2)	5.3 (10.0)
Inpatient status at time of entry, *n* (%)	2 (0.2)	3 (0.3)
PHQ-9 at week 0	18.1 (4.1)	18.8 (3.7)
PHQ-9 at week 1	14.7 (5.5)	15.9 (4.9)
BDI-II at week 1	26.2 (10.9)	28.7 (10.6)

*BDI-II* Beck Depression Inventory 2nd edition, *PHQ-9* Patient Health Questionnaire-9

Of these, 1647 had not remitted by week 3 and were individually randomised to continue sertraline (*n* = 551), combine sertraline with mirtazapine (*n* = 538) or switch to mirtazapine (*n* = 558). Participants’ characteristics proved similar across the three arms (Table 2). Of those randomised at week 3, 1613 (98.0%) and 1597 (97.0%) were successfully followed up at week 9 and week 25, respectively.

**Table 2:** Baseline characteristics of the participants at step 2 randomisation

	Continue with sertraline (*n* = 551)	Combine sertraline with mirtazapine (*n* = 537)	Switch to mirtazapine (*n* = 558)
Demographic characteristics			
Age, year mean (SD)	41.5 (11.6)	42.0 (11.7)	41.4 (11.4)
Female sex, *n* (%)	289 (52.5)	284 (52.9)	281 (50.4)
Education year, mean (SD)	14.1 (2.4)	13.8 (2.2)	14.1 (2.3)
Job status, *n* (%)			
Employed full-time	213 (38.7)	218 (40.8)	215 (38.7)
Employed part-time	52 (9.4)	52 (9.7)	40 (7.2)
On medical leave	146 (26.5)	143 (26.7)	163 (29.3)
Housewife	47 (8.5)	62 (11.6)	53 (9.5)
Student	3 (0.5)	3 (0.6)	8 (1.4)
Retired	6 (1.1)	5 (0.9)	4 (0.7)
Not employed	84 (15.3)	52 (9.7)	73 (13.1)
Missing	0	2	2
Marital status, *n* (%)			
Single, never married	188 (34.2)	144 (26.9)	188 (33.7)
Single, divorced or separated	75 (13.6)	182 (15.3)	80 (14.3)
Single, widowed	10 (1.8)	17 (3.2)	9 (1.6)
Married	277 (50.4)	292 (54.6)	281 (50.4)
Missing	1	2	0
Clinical characteristics			
Age of onset at first episode, years, mean (SD)	37.0 (12.9)	37.0 (12.8)	36.4 (12.4)
Number of previous depressive episodes, mean (SD), range	2.4 (3.4), 1–30	2.1 (3.2), 1–50	2.4 (4.2), 1–50
Length of current episode, months, mean (SD), range	5.7 (10.6), 0.5–139	6.7 (16.9), 0.5–240	6.5 (16.3), 0.5–276
Inpatient status at baseline, *n* (%)	1 (0.2)	1 (0.2)	2 (0.4)
PHQ-9 at week 3, mean (SD)	12.8 (5.2)	12.6 (5.1)	12.8 (5.2)
BDI-II at week 3, mean (SD)	24.5 (10.7)	24.1 (10.7)	24.4 (10.9)
Sertraline at week 3, mean (SD), mg/day	72.2 (26.6)	71.4 (27.6)	72.6 (28.3)

*BDI-II* Beck Depression Inventory 2nd edition, *PHQ-9* Patient Health Questionnaire-9

With regard to step 1 treatments, in the 50 mg/day arm, 91.7% (889/970) had been prescribed 50 mg/day, 0.1% (1/970) 37.5 mg/day, 1.3% (13/970) 25 mg/day and 0.1% (1/970) 75 mg/day by week 3; in the 100 mg/day arm, 82.0% (854/1041) had reached 100 mg/day, 5.3% (55/1041) 75 mg/day, 6.7% (70/1041) 50 mg/day and 0.9% (9/1041) 25 mg/day. In the 50 mg/day arm 6.8% (66/970) had stopped treatment as had 5.1% (53/1041) in the 100 mg/day arm. For step 2 randomised allocations at week 3, 99.5% (548/551), 96.1% (516/537) and 96.8% (540/558) of the randomised participants started their allocated treatment for the continuation, combination and switch arms, respectively; of the last group, 72.9% (407/558), 83.9% (468/558) and 87.8% (490/558) were successfully tapered off sertraline by weeks 5, 6 and 7, respectively.

In the following analyses, we ascertained the assumptions of normality and the homoscedasticity of the error in both the steps 1 and 2 mixed-effects models. We also ascertained the proportional hazards assumption by visual inspection of the log cumulative hazard curves and by testing the treatment*time interaction in Cox regression models.

### Step 1: 50 mg/day vs 100 mg/day as initial target dose of sertraline

According to the adjusted and weighted mixed-model repeated-measures analyses, there was no statistically significant difference (0.25 point, 95% CI − 0.58 to 1.07, *P* = 0.55) between the 100 mg/day arm and the 50 mg/day arm in the PHQ-9 score at week 9 (Table 3). There were no statistically significant differences in the secondary efficacy outcomes at week 9. Neither were there any statistically significant differences in the global burden of side effects between the 50 mg/day and 100 mg/day arms.

**Table 3:** Primary and secondary outcomes at week 9 for step 1 randomisation

	Titrate sertraline up to 50 mg/day by week 3	Titrate sertraline up to 100 mg/day by week 3	100 mg/day vs 50 mg/day
	Least squares mean (95% CI)	Least squares mean (95% CI)	Adjusted^a^ difference (95% CI) *P* value
PHQ-9	7.90 (7.14 to 8.66)	8.15 (7.78 to 8.52)	0.25 (−0.58 to 1.07) *P* = 0.55
BDI-II	16.55 (15.43 to 17.67)	16.00 (14.88 to 17.13)	−0.55 (−2.09 to 1.00) *P* = 0.49
FIBSER	5.03 (4.81 to 5.26)	5.28 (4.96 to 5.61)	0.25 (− 0.15 to 0.65), *P* = 0.22
	Raw numbers	Raw numbers	Adjusted^b^ OR (95% CI) *P* value
Proportion of response	216/424 (111 of 251 who were unremitted and allocated to continue sertraline, 91 of 129 who remitted and continued on sertraline and 14 out of 44 who withdrew from protocol)	229/425 (122 of 286 who were unremitted and allocated to continue sertraline, 84 of 99 who remitted and continued on sertraline and 23 out of 40 who withdrew from protocol)	1.23 (0.90 to 1.67), *P* = 0.19
Proportion of remission	185/424 (64 of 251 who were unremitted and allocated to continue sertraline, 110 of 129 who remitted and continued on sertraline and 11 out of 44 who withdrew from protocol)	170/426 (68 of 286 who were unremitted and allocated to continue sertraline, 86 of 99 who remitted and continued on sertraline and 16 out of 41 who withdrew from protocol)	1.09 (0.75 to 1.58), *P* = 0.64
Proportion of continuation of the allocated treatment up to week 9	302/460 (204 of 261 who were unremitted and allocated to continue sertraline, 96 of 129 who remitted and continued on sertraline and 2 out of 70 who withdrew from protocol)	330/455 (248 of 290 who were unremitted and allocated to continue sertraline, 80 of 101 who remitted and continued on sertraline and 2 out of 64 who withdrew from protocol)	1.17 (0.70 to 1.98) *P* = 0.53
	Mean (SD)	Mean (SD)	
Sertraline prescribed at week 9 (mg/day)	45.5 (19.8), *n* = 384	85.3 (32.1), *n* = 391	

^a^The linear mixed-effects repeated-measures model included fixed effects of treatment, visit (as categorical) and treatment-by-visit interaction, and random effects for subjects and sites, adjusted for age, sex, education, marital status, number of previous depressive episodes, baseline scores at weeks 0 and 1, and was weighted by inverse probability of censoring (IPCW) to account for missing outcomes due to being allocated to the combination or switching arms at step 2 randomisation

^b^We used weighted generalised linear mixed models with the logit link and binomial distributions to account for clustering effects for the dichotomous secondary outcomes, adjusted for age, sex, education, marital status, number of previous depressive episodes, baseline scores at weeks 0 and 1

*BDI-II* Beck Depression Inventory 2nd edition, *FIBSER* Frequency, Intensity and Burden of Side Effects Rating, *PHQ-9* Patient Health Questionnaire-9

At week 25, the 100 mg/day arm scored lower (− 2.28, 95% CI − 3.91 to − 0.66, *P* = 0.0059) on self-rated BDI-II than the 50 mg/day arm (Table 4). The two arms did not differ, however, in the other efficacy outcomes of PHQ-9, the proportion of remission or in the global burden of side effects.

**Table 4:** Secondary outcomes at week 25 for step 1 randomisation

	Titrate sertraline up to 50 mg/day by week 3	Titrate sertraline up to 100 mg/day by week 3	100 mg/day vs 50 mg/day
	Least squares mean (95%CI)	Least squares mean (95% CI)	Adjusted^a^ difference (95% CI) *P* value
PHQ-9	6.00 (5.33 to 6.67)	5.52 (4.89 to 6.16)	−0.47 (− 1.39 to 0.44) *P* = 0.31
BDI-II	13.29 (12.11 to 14.46)	11.00 (9.82 to 12.19)	−2.28 (− 3.91 to − 0.66) *P* = 0.006
FIBSER	4.14 (3.86 to 4.42)	4.28 (4.04 to 4.51)	0.14 (−0.20 to 0.48) *P* = 0.43
	Raw numbers (%)	Raw numbers (%)	Adjusted^b^ OR (95%CI) *P* value
Proportion of remission	240/427 (117 of 252 who were unremitted and allocated to continue sertraline, 110 of 127 who remitted and continued on sertraline and 13 out of 48 who withdrew from protocol)	226/423 (128 of 286 who were unremitted and allocated to continue sertraline, 84 of 97 who remitted and continued on sertraline and 14 out of 40 who withdrew from protocol)	0.99 (0.66 to 1.47), *P* = 0.94
	Mean (SE)	Mean (SE)	HR (95% CI) *P* value
Time to discontinuation of allocated treatment by week 25	14.68 (0.40)	13.82 (0.37)	0.88 (0.52 to 1.48) *P* = 0.63
Time to discontinuation of any treatment by week 25	17.55 (0.38)	15.55 (0.28)	1.37 (0.80 to 2.35) *P* = 0.25
	Mean (SD)	Mean (SD)	
Sertraline prescribed at week 25 (mg/day)	40.7 (29.1), *n* = 341	57.0 (45.0), *n* = 321	

^a^The linear mixed-effects repeated-measures model included fixed effects of treatment, visit (as categorical) and treatment-by-visit interaction, and random effects for subjects and sites, adjusted for age, sex, education, marital status, number of previous depressive episodes, baseline scores at weeks 0 and 1, and was weighted by inverse probability of censoring (IPCW) to account for missing outcomes due to being allocated to the combination or switching arms at step 2 randomisation

^b^We used weighted generalised linear mixed models with the logit link and binomial distributions to account for clustering effects for the dichotomous secondary outcomes, adjusted for age, sex, education, marital status, number of previous depressive episodes, baseline scores at weeks 0 and 1

*BDI-II* Beck Depression Inventory 2nd edition, *FIBSER* Frequency, Intensity and Burden of Side Effects Rating, *PHQ-9* Patient Health Questionnaire-9, *SE* Standard error

The incidence of suicidality, manic switches or serious adverse events was very small, and there were no statistically significant differences between the two arms through 25 weeks (Additional file 2: Tables S1, S2).

Results were similar regardless of baseline depression severity or initial response (Additional file 2: Table S3). All sensitivity analyses differed little from the primary findings (Additional file 2: Table S4).

### Step 2: Continue sertraline vs combine with mirtazapine vs switch to mirtazapine at week 3

The adjusted mixed-model repeated-measures analysis revealed that the mirtazapine combination arm and the mirtazapine switch arm were both superior to the sertraline continuation arm; the combination arm by − 0.99 points (95% CI − 1.55 to − 0.43, *P* = 0.0012) and the switch arm by − 1.01 points (95% CI − 1.56 to − 0.46, *P* = 0.0012) on PHQ-9 after Hochberg adjustment for multiple comparison. Results were very similar between the combination and switch arms (0.02, 95% CI − 0.54 to 0.57, *P* = 0.95); see Table 5. Self-rated BDI-II scores and the dichotomised response and remission rates were consistent with the primary analysis. RDs for response and remission, respectively, were 9.1% (95% CI 2.8–15.4%) and 12.4% (6.1–19.0%) for the combination strategy, and 8.2% (1.7–14.3%) and 8.4% (2.5–14.8%) for the switching strategy, over the continuation strategy. They corresponded with numbers needed to treat (NNTs) of 11.0 (6.5–35.7) and 8.1 (5.3–16.4) for the combination and 12.2 (7.0–59) and 11.9 (6.8–40) for the switching strategies.

**Table 5:** Primary and secondary outcomes at week 9 for step 2 randomisation

	Continue sertraline	Combine with mirtazapine	Switch to mirtazapine	Combine vs continue	Switch vs continue	Combine vs switch
	Least squares mean (95% CI)	Least squares mean (95% CI)	Least squares mean (95% CI)	Adjusted^a^ difference (95% CI) *P* value	Adjusted^a^ difference (95% CI) *P* value	Adjusted^a^ difference (95% CI) *P* value
PHQ-9	9.26 (8.79 to 9.72)	8.27 (7.80 to 8.74)	8.25 (7.79 to 8.71)	−0.99 (−1.55 to − 0.43) *P* = 0.0006 *P = 0.0012* ^b^	−1.01 (− 1.56 to − 0.46) *P* = 0.0004 *P = 0.0012* ^b^	0.02 (− 0.54 to 0.58) *P* = 0.94 *P = 0.94* ^b^
BDI-II	18.68 (17.87 to 19.49)	16.59 (15.77 to 17.40)	17.06 (16.25 to 17.86)	−2.10 (−3.12 to − 1.07) *P* < 0.0001	− 1.62 (− 2.63 to − 0.61) *P* = 0.0017	−0.47 (− 1.49 to 0.55) *P* = 0.36
FIBSER	5.34 (5.05 to 5.63)	5.43 (5.14 to 5.72)	5.59 (5.30 to 5.88)	0.09 (−0.32 to 0.50) *P* = 0.66	0.25 (−0.16 to 0.66) *P* = 0.22	− 0.16 (− 0.57 to 0.25) *P* = 0.44
	Raw numbers (%)	Raw numbers (%)	Raw numbers (%)	Adjusted^c^ OR (95% CI) *P* value	Adjusted^c^ OR (95% CI) *P* value	Adjusted^c^ OR (95% CI) *P* value
Proportion of response	233/537 (43.4%)	273/527 (51.8%)	275/550 (50.0%)	1.41 (1.11 to 1.85) *P* = 0.0055	1.39 (1.08 to 1.78) *P* = 0.0109	1.03 (0.81 to 1.33) *P* = 0.79
Proportion of remission	132/537 (24.6%)	188/527 (35.7%)	174/550 (31.6%)	1.80 (1.36 to 2.38) *P* < 0.0001	1.51 (1.14 to 1.99) *P* = 0.0037	1.19 (0.92 to 1.55) *P* = 0.19
Proportion of continuation of the allocated treatment up to week 9	452/551 (82.0%)	400/537 (74.5%)	427/558 (76.5%)	0.61 (0.45 to 0.83) *P* = 0.0014	0.68 (0.50 to 0.92) *P* = 0.0131	0.90 (0.67 to 1.19) *P* = 0.46
	Mean (SD)	Mean (SD)	Mean (SD)			
Sertraline prescribed at week 9 (mg/day)	71.7 (30.0), *n* = 520	71.1 (29.1), *n* = 502	6.5 (19.0), *n* = 524			
Mirtazapine prescribed at week 9 (mg/day)	0.6 (3.9), n = 520	15.1 (11.8), *n* = 501	18.4 (13.0), n = 524			

^a^The linear mixed-effects repeated-measures model included fixed effects of PHQ-9 at week 3, treatment, visit (as categorical), treatment-by-visit interaction and minimisation variables for step 2 randomisation (step 1 treatment, 50% or greater reduction on PHQ-9 by week 3, moderate or greater impairment on FIBSER at week 3) and random effects for individuals and sites

^b^Hochberg method was used for adjustment of multiplicity for the primary outcome (italicised *P* values for PHQ-9) but not for secondary outcomes, as postulated in the statistical analysis plan [33]

^c^We used the logistic regression model adjusted for sites, step 1 treatment, 50% or greater reduction on PHQ-9 by week 3 and moderate or greater impairment on FIBSER at week 3

*BDI-II* Beck Depression Inventory 2nd edition, *FIBSER* Frequency, Intensity and Burden of Side Effects Rating, *PHQ-9* Patient Health Questionnaire-9

The proportion of continuation of the allocated treatment was significantly lower in the combination arm (RD 8.5%, 95% CI 2.9–14.9%) and the switching arm (6.4%, 95% CI 1.3–12.5%) than the continuation arm. The overall burden of side effects as measured with FIBSER did not, however, differ among the three strategies (Table 5). Neither were there any material differences regarding incidence of suicidality, manic switches or serious adverse events (Additional file 2: Table S5).

At week 25, the treatment arms did not differ in any of the efficacy (PHQ-9, BDI-II, remission), acceptability (time to discontinuation of the allocated treatment or of any treatment) or harm outcomes (Table 6, Additional file 2: Table S6).

**Table 6:** Secondary outcomes at week 25 for step 2 randomisation

	Continue sertraline	Combine with mirtazapine	Switch to mirtazapine	Combine vs continue	Switch vs continue	Combine vs switch
	Least squares mean (95% CI)	Least squares mean (95% CI)	Least squares mean (95% CI)	Adjusted^a^ difference (95% CI) *P* value	Adjusted^a^ difference (95% CI) *P* value	Adjusted^a^ difference (95% CI) *P* value
PHQ-9	6.58 (6.09 to 7.07)	6.37 (5.88 to 6.87)	6.61 (6.12 to 7.10)	−0.20 (− 0.80 to 0.40) *P* = 0.51	0.03 (− 0.56 to 0.63) *P* = 0.91	−0.24 (− 0.84 to 0.37) P = 0.44
BDI-II	14.09 (13.15 to 15.03)	13.45 (12.49 to 14.41)	13.72 (12.78 to 14.67)	−0.64 (− 1.88 to 0.60) *P* = 0.31	−0.37 (− 1.59 to 0.86) *P* = 0.56	−0.27 (− 1.51 to 0.96) *P* = 0.66
FIBSER	4.34 (4.10 to 4.59)	4.46 (4.21 to 4.71)	4.48 (4.24 to 4.72)	0.12 (−0.23 to 0.47) *P* = 0.51	0.14 (−0.21 to 0.48) *P* = 0.44	− 0.02 (− 0.36 to 0.33) *P* = 0.92
	Raw numbers (%)	Raw numbers (%)	Raw numbers (%)	Adjusted^b^ OR (95% CI) *P* value	Adjusted^b^ OR (95% CI) *P* value	Adjusted^b^ OR (95% CI) *P* value
Proportion of remission	245/538 (45.5%)	263/520 (50.3%)	262/540 (48.5%)	1.24 (0.96 to 1.49) *P* = 0.10	1.16 (0.90 to 1.48), *P* = 0.25	1.07 (0.80 to 1.37) *P* = 0.60
	Mean (SE)	Mean (SE)	Mean (SE)	HR (95% CI) *P* value	HR (95% CI) *P* value	HR (95% CI) *P* value
Time to discontinuation of allocated treatment by week 25	15.97 (0.30)	15.23 (0.32)	15.56 (0.31)	1.07 (0.92 to 1.25) *P* = 0.40	1.04 (0.89 to 1.21) *P* = 0.64	1.03 (0.88 to 1.20) *P* = 0.70
Time to discontinuation of any treatment by week 25	17.32 (0.17)	20.47 (0.23)	20.22 (0.23)	0.89 (0.69 to 1.14) *P* = 0.35	1.08 (0.85 to 1.38) *P* = 0.51	0.82 (0.64 to 1.05) *P* = 0.11
	Mean (SD)	Mean (SD)	Mean (SD)			
Sertraline prescribed at week 52 (mg/day)	51.6 (38.5), *n* = 448	51.2 (38.3), *n* = 440	10.3 (24.7), *n* = 457			
Mirtazapine prescribed at week 52 (mg/day)	3.9 (10.6), *n* = 448	12.3 (13.0), *n* = 440	14.6 (13.8), *n* = 456			

^a^The linear mixed-effects repeated-measures model included fixed effects of PHQ-9 at week 3, treatment, visit (as categorical), treatment-by-visit interaction, and minimisation variables for step 2 randomisation (step 1 treatment, 50% or greater reduction on PHQ-9 by week 3, moderate or greater impairment on FIBSER at week 3), and random effects for individuals and sites

^b^We used the logistic regression model adjusted for sites, step 1 treatment, 50% or greater reduction on PHQ-9 by week 3 and moderate or greater impairment on FIBSER at week 3

*BDI-II* Beck Depression Inventory 2nd edition, *FIBSER* Frequency, Intensity and Burden of Side Effects Rating, *HR* hazard ratio, *PHQ-9* Patient Health Questionnaire-9, *SE* Standard error

There was no evidence of effect modification in any of the three pre-planned subgroup analyses (Additional file 2: Table S7).

## Discussion

The SUN☺D trial involved two randomisations to examine the first- and second-line antidepressant pharmacotherapies for the acute phase treatment of hitherto untreated major depressive disorder.

The step 1 randomisation examined the impact of titrating to the minimum or maximum of the licenced dose of sertraline by week 3. In patients starting treatment for major depressive disorder, there were no important differences in effectiveness or adverse effects between these two starting sertraline doses. The results of the primary outcome at week 9 (0.25 point difference in adjusted PHQ-9 score, 95% CI − 0.58 to 1.07, *P* = 0.55) excluded an important difference in favour of the higher dose. Neither were there any important differences between the minimum vs the maximum target doses in side effects, burden or treatment acceptability up to either week 9 or week 25.

When patients do not remit after 3 weeks of sertraline treatment, however, adding mirtazapine or switching sertraline to mirtazapine resulted in approximately a one-point benefit in PHQ-9 at week 9, a standardised mean difference of around 0.16. This difference corresponded to RDs in response of 9.1% (95% CI 2.8–15.4%) for the combination and 8.2% (1.7–14.3%) for the switching strategy and, in remission, of 12.4% (6.1–19.0%) for the combination and 8.4% (2.5–14.8%) for the switching strategy. These values are slightly below or above the usually accepted clinically significant threshold of 10% RD, but they may be important to patients when one considers that they are differences between alternative active treatments and that RDs for antidepressants over placebo are approximately 20% only [29, 43]. In addition, these results were consistent with patients’ self-reports by BDI-II. The benefits of combination and switching did not, however, persist after continuation treatment at the treating physician’s discretion at week 25.

Combination or switching strategies resulted in 6–8% fewer patients continuing the allocated treatments up to week 9 than continuing on sertraline. However, given the greater improvement in depressive symptoms with the strategies that included mirtazapine; similar global ratings of side effects among the treatment arms at weeks 9 and 25; and similar time to discontinuation of allocated treatment or any treatment when considered up to 25 weeks; this finding does not raise concern regarding the use of combination and switching strategies.

The most recent comprehensive network meta-analysis of antidepressants found that mirtazapine may have a greater response rate than sertraline (OR = 1.15, 95% CI 0.93–1.43) when used as first-line monotherapy [29]. The current study findings are compatible with this network meta-analysis, and this superior efficacy of mirtazapine may be responsible for some of the benefits of switching or combination strategies over sertraline continuation found in the current study.

Some guidelines recommend dose increase within the approved range after non-response to a lower dose ([4], p. 356, [10], p.53). Two previous studies have tested this strategy for sertraline and found that there was no additional benefit: one study compared 50 mg vs 150 mg of sertraline for 5 weeks after an initial 3 weeks on 50 mg and reported no difference [44]. Another study compared 100 mg vs 200 mg of sertraline for 5 weeks after an initial 6 weeks in which patients received up to 100 mg and reported that the increase to 200 mg resulted in a lower response rate [24]. Although the current study did not specifically address this strategy, our results with regard to the initial target dose are consistent with these previous studies.

There are limitations of our study. First, the step 1 cluster randomisation by clinic was performed at the start of the study. As a result, there was no concealment at the level of randomisation of individual patients, which explains the differences in characteristics of patients enrolled in the two arms. Clinicians at study sites allocated to the lower dose were less inclined to enrol severe patients or male patients than were clinicians at sites allocated to the higher dose. We dealt with this prognostic imbalance by adjusting for key variables in the mixed-model repeated-measures analyses and confirmed robustness of the primary findings against model assumptions through sensitivity analyses. Unknown confounders may still, however, have biased our findings, limiting strength of inference in the step 1 findings.

Secondly, the open-label design may have created some undetected performance bias that may threaten the internal validity of both the step 1 and step 2 comparisons, such as differential administration of co-prescriptions or psychological support. Such bias is, however, likely to be minimal, because the protocol did not allow any depression-specific psychotherapies such as CBT or IPT or concomitant administration of antipsychotics or mood stabilisers up to week 9. Co-prescription of benzodiazepines was permitted, but they were prescribed very similarly in all arms (60–70% of the participants received either anxiolytics or hypnotics). Further, a differential placebo effect associated with adding or switching to mirtazapine vs continuing with sertraline could explain some of the apparent effect of the switching/adding arms in step 2. Blinded assessment of outcomes somewhat ameliorates this concern. Moreover, the open-label design was consonant with the pragmatic nature of the study, in which we made comparisons including such possible practice variability.

Thirdly, the tapering speed of sertraline in the switching arm was relatively slow and allowed some patients to take the combination of sertraline and mirtazapine for several weeks. The efficacy of the switching strategy might therefore be contaminated by that of the combination treatment. However, gradual tapering was appropriate for this pragmatic trial and allowed us to address the impact of interventions as clinicians would implement them in the real world.

Fourthly, one might question the generalisability of the current study findings beyond the specific drugs and dosages employed. As the initial target dose of the antidepressant, we compared the minimum and the maximum of the licenced dosage in Japan, 50 mg/day and 100 mg/day of sertraline. The maximum dosage for sertraline in the USA and in some other countries is 200 mg/day. The study specified dosing for mirtazapine between 7.5 and 45 mg, which is also lower than the dosing sometimes used in other countries. These differences may reflect differences in body weights and other ethnically specific variations in the genetic as well as non-genetic mechanisms affecting the pharmacokinetics and dynamics of psychotropic drugs [45]. Up to now, however, inquiry has failed to identify ethnicity as a convincing modifier of antidepressant effect [46]. Had we compared 50 mg/day vs 200 mg/day, or had we required higher dose ranges for mirtazapine, the results might have differed. However, consistent with our step 1 findings, RCTs using other drugs have in most cases also suggested no additional benefit with higher doses [12, 47, 48]. It is also uncertain whether results would be similar had we chosen to begin with an antidepressant other than sertraline or chosen to combine or switch with a drug other than mirtazapine. Our step 2 findings for adding mirtazapine to sertraline are consistent with the previous systematic review examining various combination strategies [22]. One might consider our step 2 findings for switching to mirtazapine at odds with the recent meta-analysis on this topic that found no additional benefit in switching to various antidepressants [20]. However, the only study in this review specific to mirtazapine reported that using mirtazapine was more effective than continuing the prior medication [49].

A further limitation is that, although it provides evidence that changing therapy after failure to remit at an early point (in our study 3 weeks) may be preferable to waiting a longer period (e.g. the oft-recommended 6 to 8 weeks), the design did not specifically address the optimal timing of the decision to switch or combine. One RCT compared switching to duloxetine immediately after non-response to 4weeks of escitalopram against continuing 4 more weeks on escitalopram and then switching if non-responsive: the two arms did not differ in their primary outcome of time to response, while there was significant increase in a secondary outcome of remission in the early switch arm than in the later switch arm [50]. Another recent RCT examined the value of switching escitalopram to venlafaxine if patients have shown minimal improvement after 2 weeks; remission rates were 8% higher in the switched than in the continuation arm, but this difference did not reach statistical significance, possibly due to a small sample size (*n* = 192, *p* = 0.21) [51]. Thus, the optimal time to combine or switch remains uncertain.

Strengths of this pragmatic trial primarily relate to design features that enhance the real-world application of the results. We employed a large number of study sites using broad eligibility criteria, and thus enrolled sufficient patients to achieve high power to establish or refute differences between groups. Because Japan does not have a primary care system, patients with new onset depressive episodes usually consult office practice psychiatrists directly. Thus, in many other countries, primary care doctors would see and begin treatment of most participants entered in SUN☺D. In comparison to many multi-centre trials, we were able to enrol a large proportion (2011 of 7895) of potentially eligible patients. Enrolment of patients not yet treated for their current episode is another strength, eliminating a potential source of variability. For step 1 randomisation, we excluded patients who did not tolerate a low dose of sertraline, faithfully addressing the clinical question in which patients and clinicians are interested: In patients who tolerate an initial low dose of sertraline, should clinicians titrate to the maximum dose? For step 2 randomisation, we selected alternative interventions that are recommended in practice guidelines [4, 10] and are widely used [52].

We limited risk of bias through centralised, blinded, telephone assessments that allowed us to achieve more than 95% follow-up through 25 weeks, unique among large trials of psychiatric interventions. The centralised training of the raters enhanced the reliability and validity of the depression assessments [38]. A pre-published protocol [32], statistical analysis plan [33] and blinded interpretation of the results to minimise the researchers’ interpretation bias further enhance the trustworthiness of this RCT.

## Conclusions

The SUN☺D trial suggests that, for the initial antidepressant therapy for a new major depressive episode, titrating sertraline to the maximum over the minimum within the licenced dosage confers no additional benefit but increases cost. The confidence in this conclusion is limited by the failure of the cluster randomisation to achieve prognostic balance, and the possible residual confounding after our adjusted analysis. When patients fail to remit on this initial treatment, early combination or switching using mirtazapine resulted in a small benefit in reducing depression without an increase in adverse effects. Inferences apply to the strategies as implemented, which reflect clinical practice, including gradual tapering of sertraline in the switching arm. Factors bearing on the decision to combine or switch are likely to include costs (combination will be more costly), the current burden of side effects of the first-line treatment, the expected burden of combination or switching and the patient’s readiness to change antidepressants.

The many drugs available allow clinicians considerable options in the selection of an initial antidepressant and of second-line antidepressants to switch to or combine with. Clinicians and patients may consider starting the treatment with a low dose of agents for which evidence suggests satisfactory efficacy and acceptability in the current trial and in the comprehensive network meta-analysis [29]. Should they choose to combine antidepressants as the second-line strategy, the relative merits of potential combinations remain largely untested in RCTs, and it is implausible that RCTs will ultimately evaluate all such alternatives. A previous review suggested that a combination of a reuptake inhibitor antidepressant and an antagonist of presynaptic alpha-2 autoreceptor is more effective than other combinations [22], and the current findings were consistent with this suggestion. Those who place a high value in treatments that have been tested in large trials that both minimise risk of bias and mimic real-world conditions may prefer the specific strategies used in this trial.

## Additional files

Additional file 1: Blinded data analyses statement of interpretation. (PDF 336 kb)

Additional file 2: **Figure S1.** Schedule of the assessments. **Table S1.** Incidence of suicidality, manic switches or any serious adverse events up to week 9 for step 1 randomisation. **Table S2.** Incidence of suicidality, manic switches or any serious adverse events up to week 25 for step 1 randomisation. **Table S3.** Two pre-specified subgroup analyses for step 1 randomisation. **Table S4.** Four pre-specified sensitivity analyses for step 1 randomisation. **Table S5.** Incidence of suicidality, manic switches or any serious adverse events up to week 9 for step 2 randomisation. **Table S6.** Incidence of suicidality, manic switches or any serious adverse events up to week 25 for step 2 randomisation. **Table S7.** Three pre-specified subgroup analyses for step 2 randomisation. (DOCX 32 kb)

Additional file 3: SUN☺D Investigators and committee members. (DOCX 17 kb)
