# Supplementary material for: Optimising first- and second-line treatment strategies for untreated major depressive disorder — the SUN☺D study: a pragmatic, multi-centre, assessor-blinded randomised controlled trial
Source: BMC Med. 2018 Jul 11;16:103. doi: 10.1186/s12916-018-1096-5 (PMC6040068; doi:10.1186/s12916-018-1096-5)
Supplement: Supplementary file 1 — Blinded data analyses statement of interpretation. (PDF 336 kb) [file 12916_2018_1096_MOESM1_ESM.pdf]

Blinded data analyses statement of interpretation

SUN(^_^)D Blinded Data analyses Statement of Interpretation

The Steering and Writing Committees of the SUN(^_^)D trial (undersigned) developed and agreed on the following interpretations of the study results, based on a blinded review of the primary and main secondary outcomes data as prepared by two statisticians (one cognizant of the treatment allocations and another blinded to the treatment allocations) according to the Statistical Analysis Plan. These primary and main secondary outcomes results are appended after this signed document.

Step 1 of the study compared the minimum and the maximum licensed dosage as the initial target dose for the first-line treatment of major depressive episodes (50 mg/day vs 100 mg/day of sertraline), and Step 2 of the study compared three second-line strategies (continue sertraline, augment sertraline with mirtazapine, and switch to mirtazapine) after non-remission to the first-line treatments at week 3.

We designated two treatment arms in Step 1 as A and B and prepared two interpretations, one assuming A to be the 50 mg/day arm and B to be the 100 mg/day arm and another vice versa. We designated three treatment arms in Step 2 as X, Y and Z, and prepared six interpretations, each assuming a respective permutation of the three treatment arms.

## Step I

The cluster randomization to 50 mg/day or 100 mg/day arms, which was open, failed to achieve prognostic balance between the arms. We used the statistical adjustment to adjust for this imbalance.

### If A is 50 mg/day and B is 100 mg/day

There was no statistically significant difference in the primary outcome (adjusted PHQ-9 score at week 9, 95.8% follow-up) between the two groups (p. 3 of the results report). The same was confirmed by self-reported results by BDI-II at week 9 (p. 4). However, at week 25, the self-rated depression severity was significantly lower in the 100 mg/day arm than in the 50 mg/day arm (p. 9) while the blinded observer-ratings by PHQ-9 did not differ (p. 9); this difference however must be interpreted with caution as these were secondary endpoints after non-protocolized continuation treatment between week 9 and 25 and there were more missing data for BDI-II than for PHQ-9 at week 25. Neither were there any significant differences between the two arms in terms of response (defined as 50% or greater reduction on PHQ-9) or remission (defined as 4 or less on PHQ-9) at week 9 (pp. 4-5) or week 25 (p. 9).

In terms of acceptability, 100 mg/day was more likely to continue the allocated treatment up to week 9 (OR=1.08, p=0.0048) (p. 6). If followed-up up to week 25, however, the 50 mg/day arm was more likely to continue the allocated treatment (p. 9) (there was a crossing of survival curves between week 9 and week 13, immediately after the acute phase treatment when there were no longer any restrictions on the treatments to prescribe) (p. 42). For global ratings of side effects, suicidality, switch to mania/hypomania or serious adverse events, there were no statistically significant or patient-important differences between the two groups up to week 25 (pp. 6-8, 10-11).

The difference in the primary outcome, PHQ-9 at week 9, was an effect size of 0.15 (-0.05 to 0.35). If we assume the minimally clinically important effect size to detect between groups to be 0.2, there is little likelihood that the 100 mg/day is better than the 50 mg/day but the possibility that the 50 mg/day might be meaningfully better than the 100 mg/day was not ruled out. The results for the secondary outcome of BDI-II, the self-reported depression severity, was an effect size of -0.04 (-0.16 to 0.08) at week 9, which was precise enough to rule out any meaningful difference between the treatments. This may be due to more repeated measurements on BDI-II (at weeks 3, 5, 7, and 9) than on PHQ-9 (at weeks 3 and 9 only). The two other secondary outcomes of response and remission showed no statistically significant differences either, although for these outcomes their 95%CI did not rule out the possibility that the 100 mg/day arm might be meaningfully better than the 50 mg/day arm by an NNT of around 10.

In conclusion, in view of the fact that there were few differences between the two arms either in benefits or harms to expect up to 9 or 25 weeks, that the obtained 95% confidence interval for the primary outcome effectively ruled out the possible superiority of the 100 mg/day arm over the 50 mg/day arm for the primary outcome at week 9, and that the 100 mg/day strategy costs twice as much as the 50 mg/day strategy, we conclude that we adopt the 50 mg/day strategy as the target dose for the first-line treatment of major depression. For some secondary outcomes, the confidence intervals include the possibility of a patient-important superiority of 100 mg/day over 50 mg/day. This finding may reduce, though only to a small extent, the strength of our inference that the 50 mg/day should uniformly be chosen as the target dose for the acute phase treatment. The strength of inference is further limited by the failure of the cluster randomization to achieve prognostic balance, though that failure is mitigated by the adjusted analysis. The subgroup analyses, pre-planned and specified in the Statistical Analysis Plan, will be conducted and reported.

If A is 100 mg/day and B is 50 mg/day

There was no statistically significant difference in the primary outcome (adjusted PHQ-9 score at week 9) between the two groups. The same was confirmed by self-reported results by BDI-II at week 9. However, at week 25, the self-rated depression severity was significantly lower in the 50 mg/day arm than in the 100 mg/day arm while the blinded observer-ratings did not differ; this difference however must be interpreted with caution as these were secondary endpoints after non-protocolized continuation treatment between week 9 and 25 and there were more missing data for BDI-II than for PHQ-9 at week 25. Neither were there any significant differences between the two arms in terms of response (defined as 50% or greater reduction on PHQ-9) or remission (defined as 4 or less on PHQ-9) at either week 9 or week 25.

In terms of acceptability, the 50 mg/day arm was more likely to continue the allocated treatment up

to week 9 (OR=1.08, p=0.0048). If followed-up up to week 25, however, the 100 mg/day arm was more likely to continue the allocated treatment (there was a crossing of survival curves between week 9 and week 13, immediately after the acute phase treatment when there were no longer any restrictions on the treatments to prescribe). For global ratings of side effects, suicidality, switch to mania/hypomania or serious adverse events, there were no statistically significant or patient-important differences between the two groups up to week 25.

The difference in the primary outcome, PHQ-9 at week 9, was an effect size of 0.15 (-0.05 to 0.35). If we assume the minimally clinically important effect size to detect between groups to be 0.2, there is little likelihood that the 50 mg/day is better than the 100 mg/day but the possibility that the 100 mg/day might be meaningfully better than the 50 mg/day was not ruled out. The results for the secondary outcome of BDI-II, the self-reported depression severity, was an effect size of -0.04 (-0.16 to 0.08) at week 9, which was precise enough to rule out any meaningful difference between the treatments. This may be due to more repeated measurements on BDI-II (at weeks 3, 5, 7, and 9) than on PHQ-9 (at weeks 3 and 9 only). The two other secondary outcomes of response and remission showed no statistically significant differences either, although for these outcomes their 95%CI did not rule out the possibility that the 50 mg/day arm might be meaningfully better than the 100 mg/day arm by an NNT of around 10.

In conclusion, in view of the fact that there were few differences between the two arms either in benefits or harms to expect up to 9 or 25 weeks, and that the 100 mg/day strategy costs twice as much as the 50 mg/day strategy, we conclude that we adopt the 50 mg/day strategy as the target dose for the first-line treatment of major depression. The strength of this inference may be somewhat limited by the failure of the cluster randomization to achieve prognostic balance, though that failure is mitigated by the adjusted analysis, and that 95% confidence intervals for some outcomes included the possibility of a patient-important superiority of 100 mg/day over 50 mg/day. The subgroup analyses, pre-planned and specified in the Statistical Analysis Plan, will be conducted and reported.

## Step II

### If X is the switch-to-mirtazapine arm

After non-remission at week 3, strategies to continue on sertraline or to augment sertraline with mirtazapine were statistically significantly more effective than that to switch to mirtazapine in terms of our primary outcome (the adjusted difference -1.07, 95%CI: -1.75 to -0.38, p=0.0036 adjusted for multiplicity or -1.05, -1.73 to -0.37, p=0.0036 adjusted for multiplicity, respectively) on the blinded observer ratings of PHQ-9 at week 9 (98.0% follow-up) (p. 16). This difference translated into NNTs between 8 to 13 for response (defined as 50% or greater reduction in PHQ-9) or remission

(defined as 4 or less on PHQ-9) (p. 17). This effect was confirmed not only through the blinded rater ratings of PHQ-9 but also through patients' self-reports by BDI-II (p. 17). However, all these differences disappeared after the continuation treatment at week 25 (p. 22).

Continuing on sertraline or augmenting sertraline with mirtazapine resulted in 6-8% smaller proportion of patients continuing the allocated treatments up to week 9 than switching to mirtazapine, corresponding with NNHs between 12 to 16 (p. 21). However, there were no differences in global ratings of side effects among the treatment arms (p. 19). If examined up to week 25, there was no difference in time to discontinue the allocated treatment or to discontinue any treatment among the three arms (p. 23).

Neither were there any apparent differences in terms of suicidality, switch to mania/hypomania or serious side effects among the three arms up to 9 (pp. 19-20) or 25 weeks (pp. 24-25). Results uncorrected for multiplicity suggested that group Z (either the continuation group or the augmentation group) showed less incidence of any suicidality than the switching group (p. 19); however, we judged that this was a chance finding due to multiple comparisons and that it should not influence our conclusions.

In conclusion, we recommend the following strategies if the patient has not remitted on the first-line treatments by 3 weeks:

1) instead of switching to mirtazapine, continuing sertraline, either alone or augmented by mirtazapine, would produce a patient-important benefit corresponding with NNTs around 10 for response and remission at week 9. On the other hand, we note no patient-important differences in harms for any of these second-line treatment options.
2) the primary and the secondary outcomes from the present study suggest no difference in benefits or harms between the strategies to continue sertraline alone or to augment it with mirtazapine. Therefore we recommend, in line with many guidelines, that staying on sertraline alone up to week 9 would usually be the rational choice but that patients and their clinicians make individual judgments depending on the patients' preferences, the costs, the current burden of side effects of the first-line treatment, and the expected additional burden if it is augmented by mirtazapine.

However, the last recommendation may change depending on the results of further subgroup analyses, planned a priori, depending on the Step 1 treatments and the clinical status of the patients at week 3 (whether 50% or greater reduction has been achieved, and whether there was moderate or greater impairment due to side effects).

## If X is the augmentation arm

After non-remission at week 3, strategies to continue on sertraline or to switch to mirtazapine were statistically significantly more effective than that to augment sertraline with mirtazapine in terms

of our primary outcome (the adjusted difference -1.07, 95%CI: -1.75 to -0.38, p=0.0036 adjusted for multiplicity or -1.05, -1.73 to -0.37, p=0.0036 adjusted for multiplicity, respectively) on the blinded observer ratings of PHQ-9 at week 9 (98.0% follow-up). This difference translated into NNTs between 8 to 13 for response (defined as 50% or greater reduction in PHQ-9) or remission (defined as 4 or less on PHQ-9). This effect was confirmed not only through the blinded rater ratings of PHQ-9 but also through patients' self-reports by BDI-II. However, all these differences disappeared after the continuation treatment at week 25.

Continuing sertraline and switching to mirtazapine resulted in 6-8% smaller proportion of patients continuing the allocated treatments up to week 9 than augmenting sertraline with mirtazapine, corresponding with NNHs between 12 to 16. However, there were no differences in global ratings of side effects among the treatment arms. If examined up to week 25, there was no difference in time to discontinue the allocated treatment or to discontinue any treatment among the three arms.

Neither were there any apparent differences in terms of suicidality, switch to mania/hypomania or serious side effects among the three arms up to 9 or 25 weeks. Results uncorrected for multiplicity suggested that group Z (either the continuation group or the switching group) showed less incidence of any suicidality than the augmentation group; however, we judged that this was a chance finding due to multiple comparisons and that it should not influence our conclusions.

In conclusion, we recommend the following strategies if the patient has not remitted on the first-line treatments by 3 weeks:

1) Instead of combining two agents, continuing sertraline or switching to mirtazapine would produce a patient-important benefit corresponding with NNTs around 10 for response and remission at week 9. On the other hand, we note no patient-important differences in harms for any of these second-line treatment options.
2) The primary and the secondary outcomes from the present study suggest no difference in benefits or harms between the strategies to continue the first agent alone or to switch to mirtazapine. Therefore we recommend, in line with many guidelines, that staying on sertraline up to week 9 would usually be the rational choice but that patients and their clinicians make individual judgments depending on the patients' preferences, the costs, the current burden of side effects of the first-line treatment, the expected burden if it is switched to mirtazapine, and the readiness of each patient to completely change the antidepressant.

However, the last recommendation may change depending on the results of further subgroup analyses, planned a priori, depending on the Step 1 treatments and the clinical status of the patients at week 3 (whether 50% or greater reduction has been achieved, and whether there was moderate or greater impairment due to side effects).

If X is the continuation arm

After non-remission at week 3, strategies to augment sertraline with mirtazapine or to switch to mirtazapine after cross-tapering over 4 weeks were statistically significantly more effective than that to continue sertraline in terms of our primary outcome (the adjusted difference -1.07, 95%CI: -1.75 to -0.38, p=0.0036 adjusted for multiplicity or -1.05, -1.73 to -0.37, p=0.0036 adjusted for multiplicity, respectively) on the blinded observer ratings of PHQ-9 at week 9 (98.0% follow-up). This difference translated into NNTs between 8 to 13 for response (defined as 50% or greater reduction in PHQ-9) or remission (defined as 4 or less on PHQ-9). This effect was confirmed not only through the blinded rater ratings of PHQ-9 but also through patients' self-reports by BDI-II. However, all these differences disappeared after the continuation treatment at week 25.

Augmenting sertraline with mirtazapine and switching to mirtazapine resulted in 6-8% smaller proportion of patients continuing the allocated treatments up to week 9 than continuing on sertraline, corresponding with NNHs between 12 to 16. However, there were no differences in global ratings of side effects among the treatment arms. If examined up to week 25, there was no difference in time to discontinue the allocated treatment or to discontinue any treatment among the three arms.

Neither were there any apparent differences in terms of suicidality, switch to mania/hypomania or serious side effects among the three arms up to 9 or 25 weeks. Results uncorrected for multiplicity suggested that group Z (either the augmentation group or the switching group) showed less incidence of any suicidality than the continuation group; however, we judged that this was a chance finding due to multiple comparisons and that it should not influence our conclusions.

In conclusion, we recommend the following strategies if the patient has not remitted on the first-line treatments by 3 weeks:

1) Instead of continuing sertraline, augmenting it with mirtazapine or switching to mirtazapine would produce a patient-important benefit corresponding with NNTs around 10 for response and remission at week 9. On the other hand, we note no patient-important differences in harms for any of these second-line treatment options.
2) It is meaningful to consider the second line treatment as early as at week 3 when the patients have not remitted by then.
3) The primary and the secondary outcomes from the present study were unable to inform the decision to augment or to switch. Therefore we recommend that switching to mirtazapine would usually be the rational choice but that patients and their clinicians make individual judgments depending on the patients' preferences, the costs (augmentation strategy will be more costly), the current burden of side effects of the first-line treatment, the expected burden if it is augmented by or switched to another agent, and the readiness of each patient to completely change the antidepressant. Patients who are reluctant to give up the first medication can reasonably be offered the addition of the second.

However, the last recommendation may change depending on the results of further subgroup analyses, planned a priori, depending on the Step 1 treatments and the clinical status of the patients at week 3 (whether 50% or greater reduction has been achieved, and whether there was moderate or greater impairment due to side effects).

Kyoto, July 21, 2016

Toshi A. Furukawa, MD, PhD

Tatsuo Akechi, MD, PhD

Mitsuhiko Yamada, MD, PhD

Shiro Tanaka, PhD

Norio Watanabe, MD, PhD

Masatoshi Inagaki, MD, PhD

Yusuke Ogawa, MD, MPH, PhD

Aran Tajika, MD, PhD

Kiyomi Shinohara, MD
