# Supplementary material for: Optimising first- and second-line treatment strategies for untreated major depressive disorder — the SUN☺D study: a pragmatic, multi-centre, assessor-blinded randomised controlled trial
Source: BMC Med. 2018 Jul 11;16:103. doi: 10.1186/s12916-018-1096-5 (PMC6040068; doi:10.1186/s12916-018-1096-5)
Supplement: Supplementary file 2 — Figure S1. Schedule of the assessments. Table S1. Incidence of suicidality, manic switches or any serious adverse events up to week 9 for step 1 randomisation. Table S2. Incidence of suicidality, manic switches or any serious adverse events up to week 25 for step 1 randomisation. Table S3. Two pre-specified subgroup analyses for step 1 randomisation. Table S4. Four pre-specified sensitivity analyses for step 1 randomisation. Table S5. Incidence of suicidality, manic switches or any serious adverse events up to week 9 for step 2 randomisation. Table S6. Incidence of suicidality, manic switches or any serious adverse events up to week 25 for step 2 randomisation. Table S7. Three pre-specified subgroup analyses for step 2 randomisation. (DOCX 32 kb) [file 12916_2018_1096_MOESM2_ESM.docx]

**Additional file 2**

**Figure S1. Schedule of the assessments**

**Table S1. Incidence of suicidality, manic switches or any serious adverse events up to week 9 for Step 1 randomisation**

**Table S2. Incidence of suicidality, manic switches or any serious adverse events up to week 25 for Step 1 randomisation**

**Table S3. Two pre-specified subgroup analyses for Step 1 randomisation**

**Table S4. Four pre-specified sensitivity analyses for Step 1 randomisation**

**Table S5. Incidence of suicidality, manic switches or any serious adverse events up to week 9 for Step 2 randomisation**

**Table S6. Incidence of suicidality, manic switches or any serious adverse events up to week 25 for Step 2 randomisation**

**Table S7. Three pre-specified subgroup analyses for Step 2 randomisation**

**Figure S1. Schedule of the assessments**

|  |  |  | Week 1 | (Week 2) | Week 3 | (Week 4) | Week 5 | (Week 6) | Week 7 | (Week 8) | Week 9 | Week 13 | Week 17 | Week 21 | Week 25 |
| --- | --- | --- | --- | --- | --- | --- | --- | --- | --- | --- | --- | --- | --- | --- | --- |
| Treating physician |  | BDI-II | ● | ○ | ● | ○ | ● | ○ | ● | ○ | ● | ● | ● | ● | ● |
|  |  | PRIME-MD | ● |  |  |  |  |  |  |  |  |  |  |  |  |
|  |  | Baseline characteristics | ● |  |  |  |  |  |  |  |  |  |  |  |  |
|  |  | Suicidality/Manic switch |  |  |  |  |  |  |  |  | ● |  |  |  | ● |
|  | Site CRC | Treatment received | ● |  | ● |  | ● |  | ● |  | ● | ● | ● | ● | ● |
|  | Central rater | PHQ-9 | ● |  | ● |  |  |  |  |  | ● |  |  |  | ● |
|  |  | FIBSER | ● |  | ● |  |  |  |  |  | ● |  |  |  | ● |

BDI-II: Beck Depression Inventory-II, CRC: Clinical research coordinator, FIBSER: Frequency, Intensity and Burden of Side Effects Rating, PHQ-9: Patient Health Questionnaire-9

●: Required.

○: Optional and provided only if the patient makes the visit at that time point.

**Table S1. Incidence of suicidality, manic switches or any serious adverse events up to week 9 for Step 1 randomisation**

|  | Titrate sertraline up to 50 mg/d by week 3 | Titrate sertraline up to 100 mg/d by week 3 |
| --- | --- | --- |
| Incidence of any suicidality (C-CASA 1-7) between 0 and 9 weeks | Unremitted and allocated to continue sertraline  4/261 (1.53%),  Remitted and continued on sertraline  0/129 (0.00%),  Outside protocol treatment  2/70 (2.86%) | Unremitted and allocated to continue sertraline  9/290 (3.10%),  Remitted and continued on sertraline  0/101 (0.00%),  Outside protocol treatment  2/64(3.13%) |
| Incidence of serious suicidality (C-CASA 1-3) between 0 and 9 weeks | Unremitted and allocated to continue sertraline  2/261 (0.77%),  Remitted and continued on sertraline  0/129 (0.00%),  Outside protocol treatment  1/70 (1.43%) | Unremitted and allocated to continue sertraline  2/290 (0.69%),  Remitted and continued on sertraline  0/101 (0.00%),  Outside protocol treatment  1/64 (1.56%) |
| Incidence of mania, hypomania and mixed episodes between 0 and 9 weeks | Unremitted and allocated to continue sertraline  1/261 (0.38%),  Remitted and continued on sertraline  0/129 (0.00%),  Outside protocol treatment  0/70 (0.00%) | Unremitted and allocated to continue sertraline  1/290 (0.34%),  Remitted and continued on sertraline  0/101 (0.00%),  Outside protocol treatment  1/64 (1.56%) |

C-CASA: Columbia Classification Algorithm of Suicide Assessment

**Table S2. Incidence of suicidality, manic switches or any serious adverse events up to week 25 for Step 1 randomisation**

|  | Titrate sertraline up to 50 mg/d by week 3 | Titrate sertraline up to 100 mg/d by week 3 |
| --- | --- | --- |
| Incidence of any suicidality (C-CASA 1-7) between 0 and 25 weeks | Unremitted and allocated to continue sertraline  5/261 (1.92%)  Remitted and continued on sertraline  0/129 (0.00%)  Outside protocol treatment  3/70 (4.29%) | Unremitted and allocated to continue sertraline  11/290 (3.79%)  Remitted and continued on sertraline  0/101 (0.00%)  Outside protocol treatment  3/31 (4.69%) |
| Incidence of serious suicidality (C-CASA1-3) between 0 and 25 weeks | Unremitted and allocated to continue sertraline  3/261 (1.15%)  Remitted and continued on sertraline  0/129 (0.00%)  Outside protocol treatment  1/69 (1.43%) | Unremitted and allocated to continue sertraline  2/290 (0.69%)  Remitted and continued on sertraline  0/101 (0.00%)  Outside protocol treatment  1/64 (1.56%) |
| Incidence of mania, hypomania and mixed episodes between 0 and 25 weeks | Unremitted and allocated to continue sertraline  2/261 (0.77%)  Remitted and continued on sertraline  0/129 (0.00%)  Outside protocol treatment  0/70 (0.00%) | Unremitted and allocated to continue sertraline  3/290 (1.03%)  Remitted and continued on sertraline  0/101 (0.00%)  Outside protocol treatment  2/64 (3.13%) |
| Incidence of serious adverse events between 0 and 25 weeks | Unremitted and allocated to continue sertraline  8/261 (3.07%)  Remitted and continued on sertraline  1/129 (0.78%)  Outside protocol treatment  6/70 (8.57%) | Unremitted and allocated to continue sertraline  8/290 (2.76%)  Remitted and continued on sertraline  0/101 (0.00%)  Outside protocol treatment  8/64 (12.50%) |

C-CASA: Columbia Classification Algorithm of Suicide Assessment

**Table S3. Two pre-specified subgroup analyses for Step 1 randomisation**

1. whether the PHQ-9 score at week 1 was 15 or greater (corresponding with moderate to severe depression) or not (interaction P=0.56)

| PHQ-9 at week 9 | Titrate sertraline up to 50 mg/d by week 3 | Titrate sertraline up to 100 mg/d by week 3 | 100 mg/day vs 50 mg/day |
| --- | --- | --- | --- |
|  | Least squares mean  (95%CI) | Least squares mean  (95%CI) | Adjusted difference  (95%CI)  P-value |
| PHQ-9 score at week 1 was 15 or more (n=1150) | 10.02  (8.97 to 11.06) | 10.78  (10.05 to 11.51) | 0.77  (-0.48 to 2.01)  P=0.23 |
| PHQ-9 score at week 1 was 14 or less (n=861) | 5.60  (5.03 to 6.17) | 5.17  (4.53 to 5.81) | -0.43  (-1.32 to 0.45)  P=0.34 |

PHQ-9: Patient Health Questionnaire-9

1. whether the patient had shown improvement from week 0 to week 1 at or above the median of the sample or not (interaction P=0.87)

| PHQ-9 at week 9 | Titrate sertraline up to 50 mg/d by week 3 | Titrate sertraline up to 100 mg/d by week 3 | 100 mg/day vs 50 mg/day |
| --- | --- | --- | --- |
|  | Least squares mean  (95%CI) | Least squares mean  (95%CI) | Adjusted difference  (95%CI)  P-value |
| Had shown greater improvement by week 1 (n=847) | 6.46  (5.59 to 7.34) | 6.18  (5.53 to 6.83) | -0.28  (-1.42 to 0.85)  P=0.62 |
| Had shown smaller improvement by week 1 (n=1162) | 9.25  (8.38 to 10.11) | 9.96  (9.20 to 10.72) | 0.71  (-0.41 to 1.84)  P=0.21 |

PHQ-9: Patient Health Questionnaire-9

**Table S4. Four pre-specified sensitivity analyses for Step 1 randomisation**

|  | Titrate sertraline up to 50 mg/d by week 3 | Titrate sertraline up to 100 mg/d by week 3 | 100 mg/day vs 50 mg/day |
| --- | --- | --- | --- |
|  | Least squares mean  (95%CI) | Least squares mean  (95%CI) | Adjusted difference  (95%CI)  P-value |
| PHQ-9 according to the completers’ analysis | 8.15  (7.63 to 8.67) | 8.14  (7.70 to 8.57) | -0.02  (-0.63 to 0.60)  P=0.96 |
| PHQ-9 according to the model using linear visit instead of categorical visit | 8.34  (7.55 to 9.12) | 8.50  (8.08 to 8.91) | 0.16  (-0.70 to 1.02)  P=0.72  BIC=10354.5 |
| PHQ-9 according to the model using log(visit) instead of visit | 8.34  (7.55 to 9.12) | 8.50  (8.08 to 8.91) | 0.16  (-0.70 to 1.02)  P=0.72  BIC=10347.7 |
| PHQ-9 according to the model using actual dates for visit instead of planned weeks | 8.53  (7.76 to 9.31) | 8.63  (8.19 to 9.06) | 0.09  (-0.75 to 0.94)  P=0.83  BIC= 10223.0 |

Cf. The BIC for the primary analysis model was 10347.4.

**Table S5. Incidence of suicidality, manic switches or any serious adverse events up to week 9 for Step 2 randomisation**

|  | Continue with sertraline | Combine sertraline with mirtazapine | Switch to mirtazapine | Combine  vs Continue | Switch  vs Continue | Combine  vs Switch |
| --- | --- | --- | --- | --- | --- | --- |
|  | Raw numbers (%) | Raw numbers (%) | Raw numbers (%) | Adjusted OR  (95%CI)  P-value | Adjusted OR  (95%CI)  P-value | Adjusted OR  (95%CI)  P-value |
| Incidence of any suicidality (C-CASA 1-7) between 0 and 9 weeks | 12/551  (2.18%) | 9/537  (1.68%) | 4/558  (0.72%) | 0.80  (0.33 to 1.96)  P=0.62 | 0.29  (0.09 to 0.93)  P=0.04 | 2.73  (0.82 to 9.10)  P=0.10 |
| Incidence of severe suicidality (C-CASA 1-3) between 0 and 9 weeks | 4/551  (0.73%) | 2/537  (0.37%) | 1/558  (0.18%) | 0.56  (0.10 to 3.19)  P=0.51 | 0.24  (0.03 to 2.18)  P=0.24 | 2.38  (0.21 to 27.14)  P=0.48 |
| Incidence of mania, hypomania and mixed episodes between 0 and 9 weeks | 2/551  (0.36%) | 1/537  (0.19%) | 1/558  (0.19%) | 0.55  (0.05 to 6.42)  P= 0.64 | 0.40  (0.03 to 4.53)  P=0.46 | 1.40  (0.08 to 23.52)  P=0.81 |

C-CASA: Columbia Classification Algorithm of Suicide Assessment

**Table S6. Incidence of suicidality, manic switches or any serious adverse events up to week 25 for Step 2 randomisation**

|  | Continue with sertraline | Combine sertraline with mirtazapine | Switch to mirtazapine | Combine  vs Continue | Switch  vs Continue | Combine  vs Switch |
| --- | --- | --- | --- | --- | --- | --- |
|  | Raw numbers (%) | Raw numbers (%) | Raw numbers (%) | Adjusted OR  (95%CI)  P-value | Adjusted OR  (95%CI)  P-value | Adjusted OR  (95%CI)  P-value |
| Incidence of any suicidality (CCASA 1-7) between 0 and 25 weeks | 16/551  (2.90%) | 15/537  (2.79%) | 9/558  (1.61%) | 1.01  (0.49 to 2.21)  P=0.98 | 0.52  (0.22 to 1.21)  P=0.13 | 1.94  (0.83 to 4.57)  P=0.13 |
| Incidence of severe suicidality (CCASA 1-3) between 0 and 25 weeks | 5/551  (0.91%) | 5/537  (0.93%) | 3/558  (0.54%) | 1.07  (0.30 to 3.80)  P=0.92 | 0.57  (0.13 to 2.44)  P=0.45 | 1.88  (0.44 to 8.06)  P=0.40 |
| Incidence of mania, hypomania and mixed episodes between 0 and 25 weeks | 5/551  (0.91%) | 1/537  (0.19%) | 2/558  (0.36%) | 0.22  (0.03 to 1.95)  P=0.17 | 0.36  (0.07 to 1.93)  P=0.23 | 0.62  (0.05 to 6.97)  P=0.70 |
| Incidence of serious adverse events between 0 and 25 weeks | 16/551  (2.90%) | 12/537  (2.23%) | 14/558  (2.51%) | 0.80  (0.36 to 1.75)  P=0.57 | 0.88  (0.41 to 1.87)  P=0.74 | 0.91  (0.41 to 2.03)  P=0.81 |

C-CASA: Columbia Classification Algorithm of Suicide Assessment

**Table S7. Three pre-specified subgroup analyses for Step 2 randomisation**

1. whether 50% or greater reduction on PHQ-9 was achieved from week1 to week 3 or not (interaction P=0.18)

| PHQ-9 at week 9 | Continue sertraline | Combine with mirtazapine | Switch to mirtazapine | Combine  vs Continue | Switch  vs Continue | Combine  vs Switch |
| --- | --- | --- | --- | --- | --- | --- |
|  | Least squares mean  (95%CI) | Least squares mean  (95%CI) | Least squares mean  (95%CI) | Adjusted difference  (95%CI)  P-value | Adjusted difference  (95%CI)  P-value | Adjusted difference  (95%CI)  P-value |
| 50% or greater reduction (n=187) | 4.77  (3.77 to 5.77) | 4.97  (3.98 to 5.96) | 3.93  (2.85 to 5.00) | 0.20  (-1.10 to 1.50)  P=0.77 | -0.85  (-2.20 to 0.51)  P=0.22 | 1.04  (-0.31 to 2.39)  P=0.13 |
| Less than 50% reduction (n=1460) | 9.96  (9.30 to 10.63) | 8.65  (7.98 to 9.32) | 8.76  (8.11 to 9.42) | -1.31  (-2.06 to -0.56)  P=0.0006 | -1.20  (-1.94 to -0.46)  P=0.0015 | -0.11  (-0.85 to 0.64)  P=0.77 |

1. whether “moderate” or greater impairment due to side effects were reported on FIBSER at week 3 (interaction P=0.57)

| PHQ-9 at week 9 | Continue sertraline | Combine with mirtazapine | Switch to mirtazapine | Combine  vs Continue | Switch  vs Continue | Combine  vs Switch |
| --- | --- | --- | --- | --- | --- | --- |
|  | Least squares mean  (95%CI) | Least squares mean  (95%CI) | Least squares mean  (95%CI) | Adjusted difference  (95%CI)  P-value | Adjusted difference  (95%CI)  P-value | Adjusted difference  (95%CI)  P-value |
| “Moderate” or greater impairment (n=232) | 11.46  (10.07 to 12.85) | 8.98  (7.50 to 10.46) | 10.19  (8.75 to 11.62) | -2.47  (-4.50 to -0.45)  P=0.0169 | -1.27  (-3.27 to 0.73)  P=0.21 | -1.20  (-3.27 to 0.86)  P=0.25 |
| “Mild” or less impairment (n=1415) | 9.00  (8.34 to 9.65) | 8.11  (7.45 to 8.76) | 7.89  (7.24 to 8.54) | -0.89  (-1.61 to -0.17)  P=0.015 | -1.10  (-1.82 to -0.40)  P=0.002 | 0.22  (-0.50 to 0.93)  P=0.55 |

1. which treatment arm of Step 1 the patient was on (interaction P=0.46)

| PHQ-9 at week 9 | Continue sertraline | Combine with mirtazapine | Switch to mirtazapine | Combine  vs Continue | Switch  vs Continue | Combine  vs Switch |
| --- | --- | --- | --- | --- | --- | --- |
|  | Least squares mean  (95%CI) | Least squares mean  (95%CI) | Least squares mean  (95%CI) | Adjusted difference  (95%CI)  P-value | Adjusted difference  (95%CI)  P-value | Adjusted difference  (95%CI)  P-value |
| 50 mg/day arm (n=771) | 8.76  (7.94 to 9.59) | 8.31  (7.47 to 9.15) | 8.34  (7.52 to 9.17) | -0.45  (-1.48 to 0.58)  P=0.39 | -0.42  (-1.44 to 0.60)  P=0.42 | -0.03  (-1.06 to 1.00)  P=0.95 |
| 100 mg/day arm (n=876) | 9.91  (9.09 to 10.72) | 8.25  (7.43 to 9.06) | 8.18  (7.37 to 8.98) | -1.66  (-2.57 to -0.75)  P=0.0003 | -1.73  (-2.63 to -0.83)  P=0.0002 | 0.07  (-0.83 to 0.97)  P=0.88 |
