# Supplementary material for: Optimising first- and second-line treatment strategies for untreated major depressive disorder — the SUN☺D study: a pragmatic, multi-centre, assessor-blinded randomised controlled trial
Source: BMC Med. 2018 Jul 11;16:103. doi: 10.1186/s12916-018-1096-5 (PMC6040068; doi:10.1186/s12916-018-1096-5)
Supplement: Supplementary file 3 — SUN☺D Investigators and committee members. (DOCX 17 kb) [file 12916_2018_1096_MOESM3_ESM.docx]

**Additional file 3: SUN**☺**D Investigators and Committees members**

**Steering Committee**: Toshi A. Furukawa (Chair and Principal Investigator, Kyoto University), Tatsuo Akechi (Nagoya City University), Shinji Shimodera (Kochi University), Mitsuhiko Yamada (National Center of Neurology and Psychiatry), Masatoshi Inagaki (Okayama University), Norio Watanabe (Kyoto University), Naohiro Yonemoto (Kyoto University), Kazuhira Miki (Miki Mental Clinic), Yusuke Ogawa (Kyoto University), Nozomi Takeshima (Kyoto University), Aran Tajika (Kyoto University)

**Writing Committee**: Toshi A. Furukawa (Chair, Kyoto University), Gordon H. Guyatt (McMaster University), Tatsuo Akechi (Nagoya City University), Shinji Shimodera (Kochi University), Mitsuhiko Yamada (National Center of Neurology and Psychiatry), Masatoshi Inagaki (Okayama University), Norio Watanabe (Kyoto University), Naohiro Yonemoto (Kyoto University), Shiro Tanaka (Kyoto University), Yusuke Ogawa (Kyoto University), Nozomi Takeshima (Kyoto University), Aran Tajika (Kyoto University), Yu Hayasaka (Kyoto University), Kiyomi Shinohara (Kyoto University)

**Study Statisticians**: Naohiro Yonemoto (Kyoto University), Shiro Tanaka (Kyoto University), Qi Zhou (McMaster University)

**Data and Safety Monitoring Board**: Teruhiko Higuchi (National Center of Neurology and Psychiatry), Yoshio Hirayasu (Yokohama City University), Akiko Kada (Nagoya Medical Center)

**Site Principal and Co-principal Investigators**: **Hokkaido University**: Tsukasa Koyama, Ichiro Kusumi, Takeshi Inoue, Yuki Kako, **University of Tokyo**: Kiyoto Kasai, Motomu Suga, **Toho University**: Masafumi Mizuno, Naohisa Tsujino, **Nagoya City University**: Tatsuo Akechi, Masaki Kondo, **Hiroshima University**: Shigeto Yamawaki, Yasumasa Okamoto, **Kochi University**: Shinpei Inoue, Shigeru Morinobu, Shinji Shimodera, **Kurume University**: Naohisa Uchimura, Shingo Yasumoto, Keiichiro Mori, **Kumamoto University**: Manabu Ikeda, Noboru Fujise

**Participating Clinical Sites** (number of participants recruited):

**Hokkaido University** (121): Kahori Itoh (Sinsapporo Mental Clinic), Kunihiko Kawamura (Kawamura Mental Clinic), Takeshi Inoue (Hokkaido University/Tokyo Medical University), Masahito Tsukamoto (Odorinishi 11-chome Kokorono Clinic), Miki Noda (Sapporo Hanazono Hospital/Maki Hospital), Ikuko Nitta (Konohana Mental Clinic), Shin Nakagawa (Hokkaido University), Koichi Ito (Sappro Hanazono Hospital), Teruaki Tanaka (Hokkaido University/Kushiro City General Hospital), Yukie Usukubo (Sapporo Hanazono Hospital), Tomoko Fujie (Sapporo Hanazono Hospital), Yuki Kako (Hokkaido University), Ihoko Suzuki (Sapporo Kokoronomori Clinic)

**University of Tokyo** (174): Kazuyuki Sugishita (Oji Mental Clinic), Naohiro Okada (Higashi Omiya Mental Clinic/University of Tokyo), Masaru Kinou (Ohara Clinic), Kohei Marumo (Hongo-Todaimae Kokorono Clinic), Makoto Motoyama (Miyahara Mental Clinic), Eisuke Sakakibara (Higashi Omiya Mental Clinic/University of Tokyo), Kazuki Taniguchi (Higashi Omiya Mental Clinic), Fumichika Nishimura (Toyocho Kokorono Clinic/University of Tokyo), Mariko Tada (Toyocho Kokorono Clinic/University of Tokyo), Shinjiro Dogan (Toyocho Kokorono Clinic), Shuntaro Ando (Hongo-Todaimae Kokorono Clinic/University of Tokyo), Norichika Iwashiro (Hongo-Todaimae Kokorono Clinic/University of Tokyo)

**Toho University** (157): Bun Chino (Ginza Taimei Clinic), Kazuhira Miki (Miki Mental Clinic), Naohisa Tsujino (Toho University), Yuki Kato (Toho University), Sayaka Aikawa (Toho University), Michiko Nakamura (Toho University), Hiroko Hasuya (Toho University)

**Nagoya City University** (601): Tadashi Kato (Aratama Kokorono Clinic), Yoshio Ikeda (Narumi Himawari Clinic), Yoshihiro Shinagawa (Shiki Clinic), Masaki Kondo (Aratama Kokorono Clinici/Nagoya City University), Yoshiyuki Itakura (Itakura Clinic), Ushio Isobe (Isobe Clinic), Taku Sugiura (Aratama Kokorono Clinic/Inuyama Hospital), Toshiyuki Watanabe (Watanabe Mental Clinic), Mino Kayukawa (Aratama Kokorono Clinic), Norio Watanabe (Nagoya City University/Kyoto University), Tatsuo Akechi (Nagoya City University), Yumi Nakano (Nagoya City University)

**Hiroshima University** (369): Akio Mantani (Mantani Mental Clinic), Ken’ichi Kurata (Kabe Mental Health Clinic), Ichiro Yanai (Yanai Mental Health Clinic), Kyoka Ozaki (Morioka Clinic), Hiroaki Izumi (Izumi Clinic), Yasuyuki Iwamoto (Yamasaki Clinic), Yukitaka Morita (Hiroshima Citizens Hospital/Morita Mental Clinic), Ken Wada (Hiroshima Citizens Hospital)

**Kochi University** (325): Hajime Kubouchi (Kokokara Clinic), Hirotoshi Sato (Harimayabashi Clinic), Shinji Shimodera (Kochi University), Hirokazu Fujita (Kochi University), Takashi Itoh (Medical Counseling Room Itoh Clinic), Ryosuke Fujito (Fujito Hospital), Sawako Kanno (Atago Hospital), Hiroshi Hashizume (Fujito Hospital), Ippei Morokuma (Kochi University), Yosuke Suga (Kochi University), Mikako Fuji (Kochi University)

**Kurume University** (161): Susumu Hirota (Hirota Clinic), Ken Uematsu (Kurume University), Shigeto Yamada (St. Lucia Hospital), Yuuki Hanada (Kurume University), Yuji Ito (Kurume University), Hiroshi Chikama (Kurume University), Hidetsugu Kodama (Kodama Hospital), Keiichiro Mori (Kurume University), Takehiro Morita (Kurume University), Misari Oe (St. Lucia Hospital/Kurume University), Shingo Yasumoto (Kurume University), Taro Oji (St. Lucia Hospital), Hidefumi Kodama (Kodama Hospital), Kimihiro Ogi (Kurume University), Yoshihisa Shoji (Kurume University)

**Kumamoto University** (103): Noboru Fujise (Kumamoto University), Shigehiro Nakata (Yoyasu Clinic), Masahiro Shono (Yuge Hospital), Akinori Aizawa (Yuge Hospital), Kosuke Nishiyama (Yuge Hospital), Yasuhisa Abe (Yatsushiro Kosei Hospital), Midori Suematsu (Yuge Hospital), Ken Ikegami (Ikegami Clinic), Kazuya Isoda (Yuge Hospital), Kenshiro Miyamoto (Yatsushiro Kosei Hospital), Setsuko Yasugawa (Yatsushiro Kosei Hospital), Erika Mizutani (Yuge Hospital), Ryuta Fukunaga (Kumamoto University/Yatsushiro Kosei Hospital), Yoshitomo Nishi (Kumamoto University)
